# Sustainable Monitoring and Surveillance Systems to Improve HIV Programs: Review

**DOI:** 10.2196/publichealth.8173

**Published:** 2018-04-24

**Authors:** Daniel Low-Beer, Mary Mahy, Francoise Renaud, Txema Calleja

**Affiliations:** ^1^ World Health Organization Geneva Switzerland; ^2^ UNAIDS Geneva Switzerland

**Keywords:** surveillance, HIV, development monitoring, evaluation

## Abstract

HIV programs have provided a major impetus for investments in surveillance data, with 5-10% of HIV program budgets recommended to support data. However there are questions concerning the sustainability of these investments. The Sustainable Development Goals have consolidated health into one goal and communicable diseases into one target (Target 3.3). Sustainable Development Goals now introduce targets focused specifically on data (Targets 17.18 and 17.19). Data are seen as one of the three systemic issues (in Goal 17) for implementing Sustainable Development Goals, alongside policies and partnerships. This paper reviews the surveillance priorities in the context of the Sustainable Development Goals and highlights the shift from periodic measurement towards sustainable disaggregated, real-time, case, and patient data, which are used routinely to improve programs. Finally, the key directions in developing person-centered monitoring systems are assessed with country examples. The directions contribute to the Sustainable Development Goal focus on people-centered development applied to data.

## Introduction

The United Nations (UN) Sustainable Development Goals (SDGs) launched in 2015 are a universal set of 17 goals, targets, and indicators that United Nations member states are expected to use to frame their agendas and political policies. These SGDs have consolidated HIV, tuberculosis (TB), malaria, and other communicable diseases into one target, Target 3.3, and health into one goal, Goal 3 [1,2].

At the same time, data have increasing prominence in the SDGs and are seen as one of the three systemic issues in implementation (Goal 17) [3]. There are two data targets, for (1) reliable, timely, disaggregated data (Target 17.18) and (2) capacity for data use, namely statistical and analytic capacity for countries to measure their own progress (17.19).

Data were largely highlighted for reporting against targets in the UN’s Millennium Development Goals (MDGs) of 2000. In the SDGs, routine data use has a much more active, ongoing role as part of implementation—the so-called data revolution. The shift in the role of data from the MDGs to the SDGs is from reporting after implementation, to using data upfront as part of how development is implemented. As the Independent Expert Advisory Group report on Mobilising the Data Revolution for sustainable development states, “Data are the lifeblood of decision-making and the raw material for accountability” [4].

In fact, HIV programs have made major contributions to investments in data; for example, the US President’s Emergency Plan for AIDS Relief (PEPFAR) program has supported population-based surveys with, more recently, surveys including incidence measurement. The Global Fund to fight acquired immune deficiency syndrome (AIDS), TB, and malaria recommends that 5-10% of its US $4 billion funds each year is invested in monitoring and evaluation (M&E) and has specific strategic initiatives to invest in district health information systems and key population data [5]. However, the SDG priority to use data routinely leads to three major directions for the surveillance agenda.

First, countries need to develop sustainable, routine patient and case surveillance systems based on program records since measures of prevalence from national population-based surveys are prohibitively expensive for most countries.

Second, although there are 100 indicators defined by the World Health Organization (WHO) for health, there are only five cross-cutting data systems, which generate the majority of these data [6]. There is increasing agreement among partners to align support to the basic data systems, where data can then be used to improve programs. Alignment of partners to support data systems may be more effective than the MDG focus on agreeing indicators.

Third, there is a need for more disaggregated, individual level data, which can be used for ongoing program improvement. Routine data systems can be used for both patient care and the majority of surveillance needs [7-9].

This paper reviews an important shift towards routine monitoring and surveillance systems in the context of the wider M&E of the HIV response. This paper reviews the global surveillance priorities agreed by HIV partners in 2015 at the onset of the SDGs [10]. Second, the increasing use of sustainable, routine data systems for health care and surveillance needs is highlighted. Finally, the key directions to develop sustainable surveillance systems over the next 5 years, including person-centered case surveillance and patient monitoring systems, are discussed [11].

## A Common HIV Surveillance Agenda: Towards Sustainable Data Systems

In 2015, the WHO and the Joint United Nations Programme on HIV/AIDS (UNAIDS) held the 3^rd^ Global Consultation Meeting on HIV surveillance aiming to review country surveillance needs, assess priority gaps, and consolidate a global surveillance agenda from 2015 to 2020 [10]. This country and partner meeting coincided with the shift from the MDGs to the SDG agenda.

**Figure 1:**
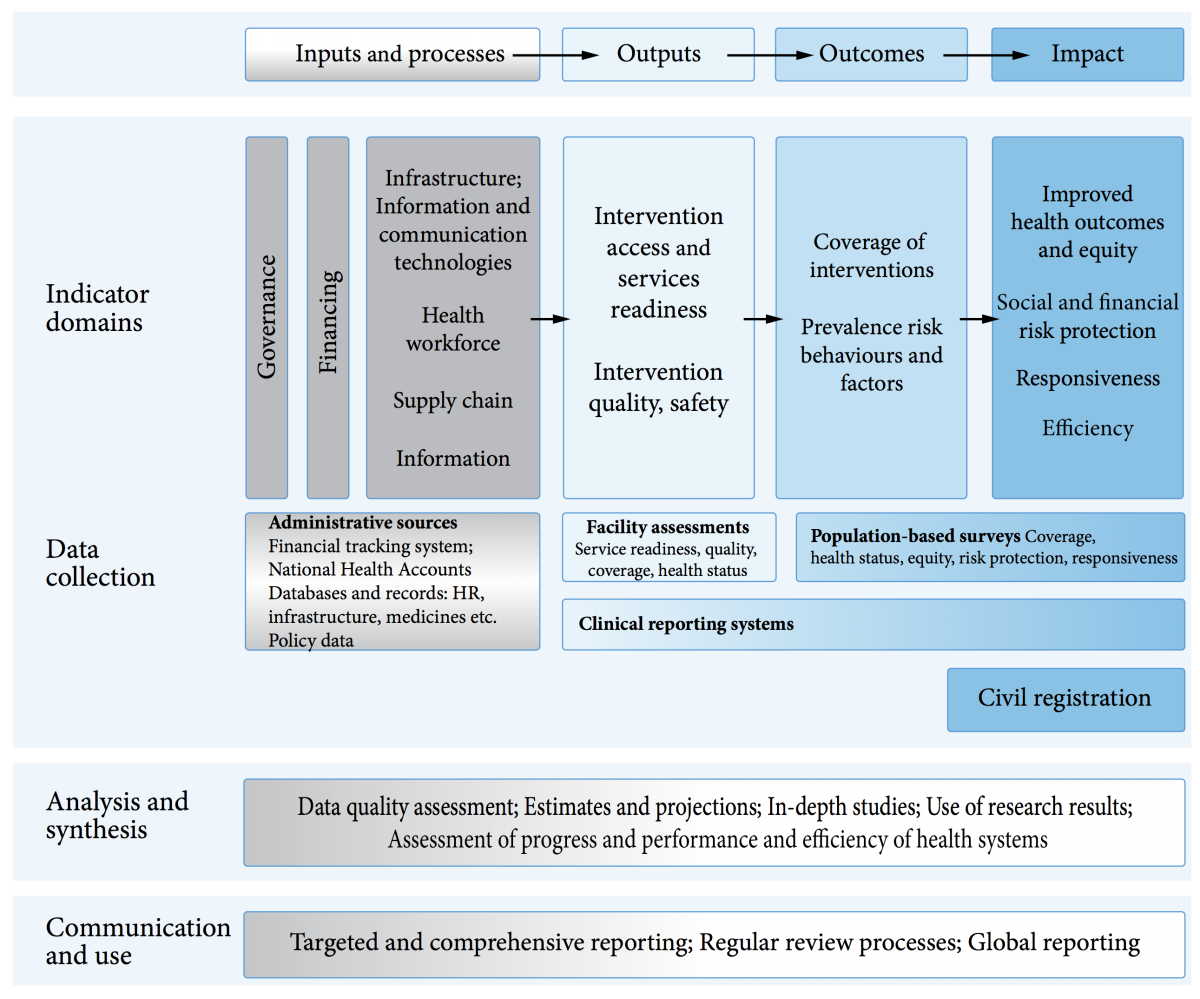
Common M&E framework defined by WHO.

The WHO country-led platform for information and accountability [12] highlighted that underlying the many indicators, there were five key data collection systems (Figure 1). These were identified as population-based surveys, facility assessments, administrative sources, clinical reporting systems, and civil registration. The data systems provided a more consolidated focus than indicators for partner alignment in monitoring and surveillance.

A critical balance is required between the periodic use of population-based surveys and the more routine systems for measuring facility, clinical, and civil registration (and administrative) events [13]. Initially, AIDS case surveillance had ensured data on diagnosis were collected in facilities as part of health workers’ ongoing patient care, supporting direct engagement with people living with HIV and care from their families and communities. This routine data played an important role in early community and prevention responses [14,15]. Increasingly, HIV surveillance data have been taken out of the health settings where the diagnosis and care of individuals occurs.

In 2015, it was important to rebalance support of all five components of data collection in the context of the SDGs. The global HIV surveillance agenda was developed by partners to support a shift towards more sustainable surveillance. It also highlighted some key gaps and challenges, including for key populations, to directly measure HIV incidence, to standardize and improve the quality of routine individual-level data, and to use routine data more actively for program improvement.

The country and global partners agreed on the role of surveillance data and a shift in balance towards routine, integrated surveillance data at a local level, based on the following principles:

1. A key component of ending AIDS epidemic is better quality, local, granular, and disaggregated data to design and support a sustainable response.
2. Supporting the health services cascade requires a cascade of linked data.
3. The use of surveillance data is an intervention in itself allowing programs and communities to better respond to the epidemic with services.
4. Surveillance requires systematic investments of at least 5-10% of program funds so that overall funds are focused on the epidemic and so that impact can be assessed.
5. Increased support is needed for routine, integrated, district health data as part of health information systems including sexually transmitted infections and hepatitis, linked to real-time health decisions.

After reviewing the evidence of developments in surveillance methods and country implementation, five key surveillance priorities were agreed on at the Global Surveillance Meeting in 2015, set for the period 2015-2020 [10]):

1. HIV prevalence (and incidence) data that are granular and disaggregated to the local level, by age, sex, and populations
2. Key population data that inform program and national estimates, including stigma, behaviors, and linkage to services
3. HIV case reporting and facility data that monitor coverage and linkages from prevention to treatment and to other health areas
4. Measurement and review of mortality and incidence, including modeling and estimation approaches, and program impact reviews
5. Analysis capacity to use multiple large datasets from various sources, including data from surveys, facilities and communities, and from new media, for advocacy, program improvement, and estimating impact

Surveillance priorities for 2015-2020 were agreed on that described the guidance required and status of implementation (Table 1). This provides an aligned partner and country approach to surveillance data in HIV, which has often been fragmented [6,8]. In addition, regular epidemiological reviews provide an input to wider country program reviews, providing a greater focus on impact.

**Figure 2:**
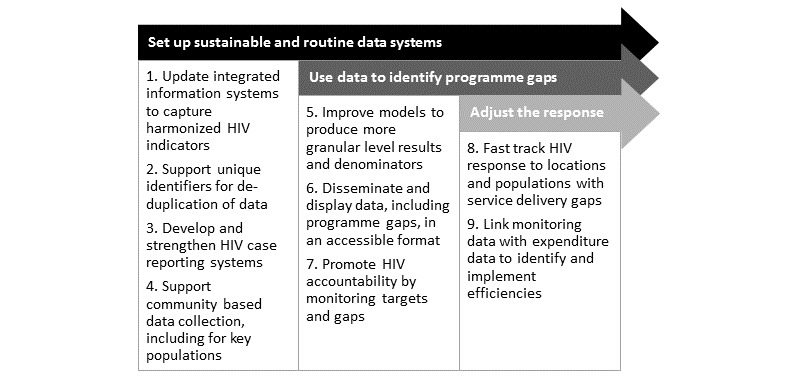
Shift in strategic information to fast track the HIV response (developed by UNAIDS and supported by WHO).

Based on the global surveillance agenda, WHO and UNAIDS have increased support for sustainable routine data systems and use [16]. To achieve the fast track targets, UNAIDS has proposed a shift in strategic information systems (Figure 2). The first four elements of Figure 2 shows the foundation for a sustainable routine monitoring system described in this paper.

The proposed shift in strategic information strengthens the focus on community-based data collection and links to finance and expenditure data. By analyzing the granular routine data with expenditure data and modeling where required, countries will have the strategic information necessary to fast-track the HIV response in the geographic areas and populations that are most in need. [17].

**Table 1:** Global surveillance priorities and work plan for development.

Key area	Guidance status	Gaps to fill
1. Incidence	Yes, for household surveys, gaps for routine data	Guidance on incidence assays for surveys
		Application for case diagnoses
2. Mortality	Yes, for civil registrations and vital statistics, and for demographic sentinel surveillance sites	Guidance on sentinel or routine data on HIV-related mortality
3. Household surveys	Guidance provided	Need for update on household surveys
4. Key population data	Yes	Bio-behavioral surveys, guidelines to complete
		Strategic framework for use of data on key populations, from program to national levels
		Guidance on size estimate algorithm, use for local and national programs
5. Case surveillance	Yes, in draft	Guidance on person-centered monitoring, patient and case surveillance, and use of unique identifiers in HIV and health
6. ANC routine testing data	Shift from antenatal clinic sentinel sites to prevention of mother to child transmission of HIV and use of routine testing data	Gap for implementation support for use of testing data for surveillance and also for new infections
7. Analysis capacity	Yes, need to support cascade gap analysis	Impact reviews and prioritization: new sources of impact data
		Big data: new analysis methods for facility and program data

## Developing Routine Monitoring Systems for Patient Care and Surveillance

WHO has convened partners including Centers for Disease Control and Prevention (CDC), UNAIDS, Global Fund, civil society, and countries to consolidate guidance on person-centered patient monitoring and case surveillance data. This aims to consolidate routine data systems to be used for improved care and for the majority of reporting needs.

The aim of this guidance is to combine routine, real-time patient and program monitoring and surveillance into a consolidated M&E system. This involves defining standardized sentinel events (from diagnosis, treatment to viral suppression, and outcomes, which are reported in the same way across a program) for consistent reporting. The guidelines extend this surveillance system from patients, to all cases diagnosed with HIV, and with unique identifiers to link to other health services.

The development of these guidelines was based on a country situation analysis of sustainable country monitoring systems at different levels of development, regions, and contexts (Table 2).

The guidelines define a sustainable routine monitoring system based on (1) promoting the use of routine data for patient care and enabling reporting on most program, national, and global indicators in a sustainable manner, (2) supporting linkage of HIV patient care to wider health care needs and monitoring, including mortality data, through the use of unique identifiers and the principles of interoperability of data systems, (3) building on ongoing, routine monitoring systems in countries to strengthen country capacity, and (4) providing disaggregated, person-centered data to improve services and to support the ongoing, real-time use of data at local level for improved health care and program improvement.

While developing patient monitoring systems improve patient health care, major risks of confidentiality and security of data also need to be assessed in each context. The guidelines aim to strengthen investments in the robustness, interoperability, and security of routine data systems. The approach produced in the recent WHO guidelines is in line with the SDGs on people-centered development and “leaving no one behind,” where data are disaggregated to ensure that, for example, key populations and groups by age, gender, and geography receive services.

**Figure 3:**
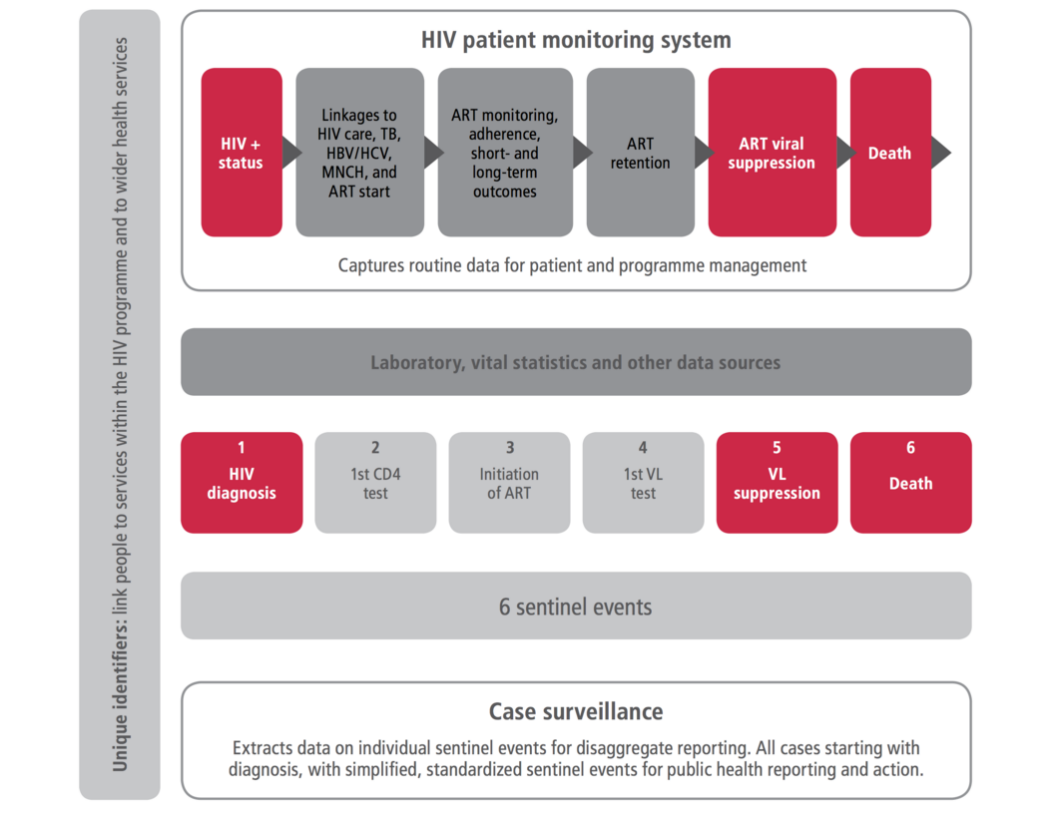
A consolidated routine M&E system for HIV patient monitoring and surveillance of key program measures (yellow boxes show existing indicators in patient monitoring, red shows measures requiring considerable additional investment to improve and adjust data systems).

The approach to strengthening routine data for patient monitoring and surveillance consists of three key components, as presented in the guidelines on person-centered monitoring: (1) use of standardized processes and tools for HIV patient monitoring at the health facility level, linked to priority indicators for national and global level reporting, (2) strengthening of case-based surveillance for HIV to integrate all cases of HIV based on key sentinel events starting with diagnosis, and (3) use of unique identifiers to link patients and their related health data between health services, for example, to link people with HIV on lifetime treatment to wider chronic care services (Figure 3).

**Table 2:** Situation analysis of countries developing person-centered case surveillance and patient monitoring.

Country	Surveillance system	Program improvements and gaps
Haiti	Individual case surveillance introduced with single national dataset integrating multiple sources. Data de-duplicated and used to identify transfers. Minimal cost, as built on existing infrastructure and data.	Targeted HIV treatment services as populations migrated seasonally. Better directed prevention resources. Generates routine reporting.
Zimbabwe	Building case surveillance on patient monitoring system. 80% of records contain unique identification of national insurance number. Need to invest in a robust and secure macro database to link facilities.	Major benefits for retention and contacting those lost to follow-up, removing those who have gone to other facilities or who have died. Need to invest in a robust and secure macro database to link facilities.
Brazil	Primary case reporting in place built for payment purposes, not surveillance. Labs require CD4 and viral load to receive payment from Ministry of Health. Uses names and includes key population information to assess equal access.	Works well and improves follow-up and payment. Major limitation does not include private laboratories. Assess access to key populations, ensure confidentiality and human rights protection.
Zambia	Smartcard system used to link patient records but does not cover all facilities. Not all facilities linked online; data collected on memory sticks from some sites.	Major benefit of being able to de-duplicate testing and treatment records, for improved patient management and more accurate reporting.
Malawi	Health “passport” for all health services. Differentiated system in which all HIV sites with more than 2000 patients use electronic medical records, but most sites are still paper-based. Data are entered into electronic database centrally.	Quarterly reporting from routine system for management, and major benefits for drug forecasting. Next step to integrate HIV with national identification and health passport.
Thailand	Unique identification based on social insurance, links key databases for patient management.	Improved availability and speed of lab test results, improved reimbursement. Migrants not covered by national unique identification.
Botswana	Routine use of national unique identification and insurance number for access to all HIV, health, and social services	Easier access, transfer and linkage to a range of HIV and health services.
Western Cape, South Africa	Three-tiered system with paper at lowest level, entered into electronic register at district level, and electronic records in 15 sites. Tier.net in 3000 sites which feeds back to patient management.	Regular, routine reports to facilities on loss to follow-up, viral load data to improve patient care and de-duplicate data.
Myanmar	Patient reporting system initially based on non-governmental organization (NGO) programs delivered by Médecins Sans Frontières. Challenge is transition to national system with investments in patient index, interoperability and links to health information system software.	Strong data on treatment cascade routinely used to highlight gaps and improve late initiation of antiretroviral therapy (ART). Facilitates planning and global reporting.

**Figure 4:**
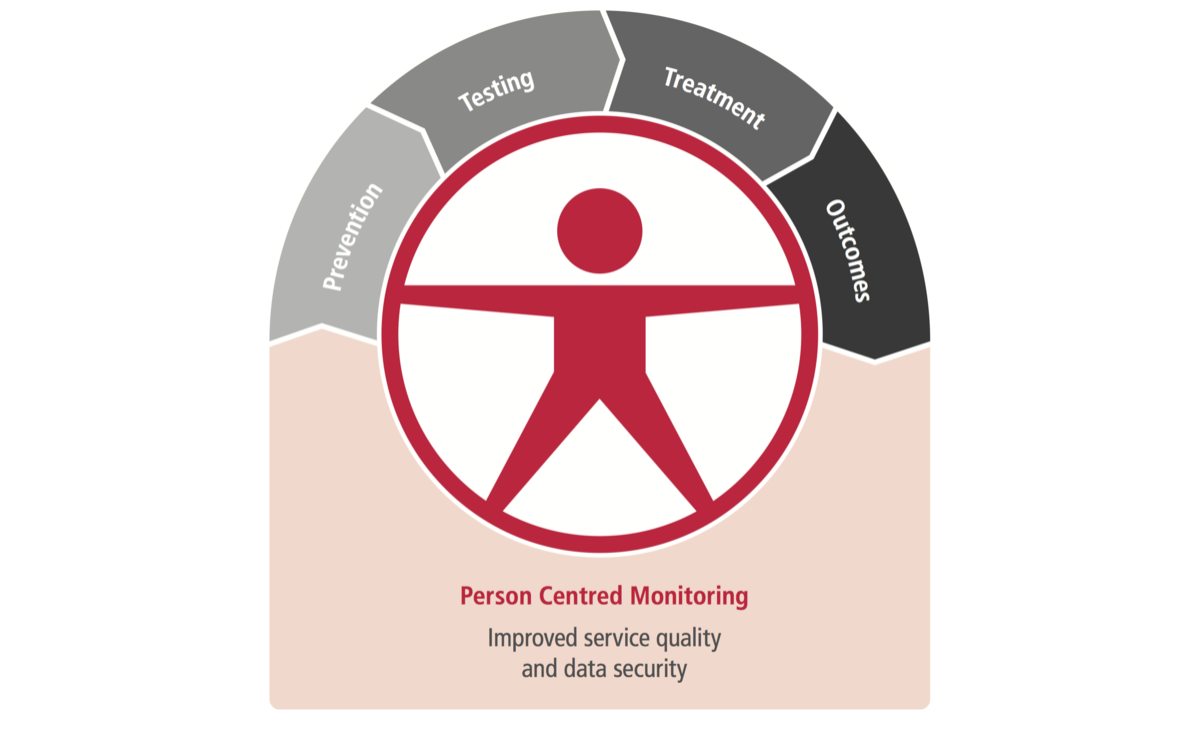
Key data sources to support reporting on the cascade of services.

A consolidated, routine data system needs to link data from different sources focused on data use (Figures 1, 3 and 4 ). This is facilitated by the introduction of unique identifiers so different data can be consolidated to support the person receiving services over their lifetime (and provide the basis for chronic care for HIV and wider healthcare). The data can provide routine information on most indicators for HIV reporting.

However, further work is needed to strengthen the reporting on community data, key populations, and prevention services provided outside facilities to inform and guide country-led prevention and control efforts. There are innovative approaches in a number of countries to link community and facility data into a single reporting system, for example in Kenya for prevention, in Zimbabwe linking sex worker outreach data to initiation of care, in India with community prevention mapping, and in Cambodia to focus on HIV incidence reduction between prevention and treatment. However, this area requires considerable strengthening as identified by UNAIDS [12,18].

The guidelines that WHO, UNAIDS, CDC, PEPFAR, and Global Fund support provide the basis of a sustainable routine, person-centered M&E system. The guidelines provide 15 recommendations to develop this approach, based on maturing and strong investments in existing country data systems, data security, and analytical capacity in countries (Table 3).

The guidance provides the backbone for sustainable routine monitoring and surveillance systems.

**Table 3:** Recommendations for person-centered patient monitoring and case surveillance.

Recommendations			Supporting tools (online annexes)
**Patient monitoring**			
1.	Collect a minimum, standardized dataset for patient care.		Guidance on a minimum dataset for patient monitoring.
2.	Transition monitoring to “treat all”: Depending on national guidelines, countries should transition from using the pre-ART register to using the ART register.		Guidance for this transition.
3.	Simplify and standardize tools (cards, registers, and reports) across facilities.		Generic tools for adaptation.
4.	Integrate and link HIV and health reporting; the HIV card should form part of the patient folder or passport integrated with primary health.		Generic HIV patient card and ART register for country adaptation.
5	Implement regular data quality reviews and invest in data use.		Guidance on carrying out an annual patient monitoring review and improving quality of care.
**Case-based surveillance**			
1.	Standardize reporting of sentinel events: Standardized sentinel events should be identified to include the 6 key sentinel events (HIV diagnosis, first CD4 test, initiation of ART, first viral load test, viral load suppression, mortality).		Definitions of six key sentinel events
2.	De-duplicate testing and treatment data to support facilities and improve data quality: Case-based surveillance should provide de-duplicated counts of diagnosed persons and people on treatment for reporting and to be shared with facilities.		Guidance on approaches.
3.	Develop case surveillance based on a country situation analysis. Improvements to case-based surveillance should be based on a country situation analysis that identifies and costs incremental improvements, and not introduced as a separate monitoring approach.		Tool for country situation analysis.
4.	Start case surveillance with HIV diagnosis and build on patient monitoring.		Guidance on HIV case definitions and case surveillance; requires reporting on HIV diagnosis in addition to and linked to treatment data.
5.	Ensure confidentiality and security of all data, particularly for key population data. The guidance suggests that risk behavior and key population data be assessed at the point of diagnosis and to support referral to care. However, it is not routinely included in patient monitoring, where there are risks.		Recommendations on key population data
**Scaling-up unique identifiers for person-centered monitoring**			
1.	Introduce and use unique identifiers for data shared across a program.		Definitions and examples of unique identifiers.
2.	Transition progressively from paper-based to electronic patient information systems. Countries should use a tiered approach starting with high volume sites.		Example of a tiered approach.
3.	Strengthen and differentiate data security: significant investments are now required in databases and policies to protect and differentiate security and confidentiality of key data.		Guidance on key components of strengthening and differentiating data security
4.	Invest in data systems and promote interoperability and open source standards.		5-10% of program budgets are used to strengthen monitoring and evaluation.
5.	Use data to improve programs, to strengthen retention, linkage and transfer. Data use drives data and program improvement.		Investments in data analysis functions and dashboards, which feedback data and can convene and measure program improvements.

## Routine Community Data for Key Populations

Integrating community level data into district, province, and national surveillance and monitoring systems is fundamental for shifting to sustainable routine data systems. Community-based organizations provide care and treatment services for key populations linked to essential prevention activities. The provision of these services are an important component of the HIV response in a country and must be considered when identifying populations not reached by prevention and treatment services.

At the program level, key population surveillance systems can benefit from many of the components of routine data systems. However, if the policy and data environments are not protective, it may be safer for this data to be kept at NGO and program level for the benefits of delivering services. In this case, no individual level data from the key population services will be available in the national monitoring system.

Further work is needed to strengthen the reporting of community data on key populations and prevention services provided outside of facilities. Routine data must be supplemented by regular surveys to assess HIV prevalence, incidence, and behavior to provide insight into whether the data collected in the routine systems are representative.

## Routine HIV Prevalence Data From Antenatal Clinics

Since the early 2000s, UNAIDS and WHO have recommended that countries conduct sentinel surveillance among pregnant women attending antenatal clinics to determine trends in HIV over time. However these surveys require personnel and financial resources that are not often available without outside funding. These surveys were conducted every 2 years and were usually targeted at high-volume antenatal clinics often in urban areas. These data were critical for understanding the trajectory of the HIV epidemic historically.

As more countries started testing all pregnant women attending antenatal clinics, these routinely collected data have started to be used for surveillance purposes. Since 2017, it is possible to include these routine data in the modeling software that countries use to produce estimates of HIV incidence, prevalence, and AIDS-related deaths. These data allow for more granular models and allow countries to produce estimates every year instead of every 2 years.

## Priority Actions and Investments in Data

The Global Surveillance Plan, 2015-2020 (Table 1) identified the following investment priorities to strengthen surveillance systems:

1. The strengthening of incidence and mortality methods and data, which can be used or with mortality data linked to routine surveillance
2. Strengthening of key population M&E, including surveys and size estimation. Many of the benefits of routine data in key populations are even more valuable for services and program management. However, issues of confidentiality and data security can be even more critical in these populations in certain settings.
3. Investing in analysis capacity, so that data are used for program improvement. The consolidated strategic information guidelines also called for data analysis positions or a small team in the Ministry of Health to respond to the demand for data for program improvement.
4. Support transition to using routine HIV testing data from antenatal clinics for routine prevalence surveillance. This is a key component of routine surveillance for all diagnosed cases of HIV and to develop approaches to measure HIV prevalence from this data.
5. In addition, periodic household surveys will still be important to supplement routine monitoring data, particularly in a limited number of high-burden countries. However, they should be supplemental rather than replace the use of sustainable routine surveillance data in most countries.

### Conclusion

The context of the SDGs provides both impetus and new directions for the surveillance agenda. There is a greater emphasis on data as a major component of implementation, at a time when HIV funding from donors is no longer increasing.

First, there is a shift towards more routine, disaggregated, real-time data. Routine data will be supplemented by national population-based surveys with biomarkers in a limited number of high-burden countries. Strong information systems based on routine data will allow further disaggregation of data (at least by age, sex, and geographic area) in accordance with SDG target 17.18.

Second, investments will need to be made in analytical capacity to better standardize, communicate, and use these data to improve programs. The value of data is in its use to improve programs at national, subnational, facility, and community level. This requires the human capacity for analysis and use alongside the hardware of M&E systems.

Finally, the value of consolidated, sustainable routine surveillance systems is for reporting but also for ongoing, real-time improvements to programs. In the coming few years, the evidence for the benefits of data as an intervention in its own right will need to be collected. This will be key to the medium-term sustainability of these remaining routine monitoring and surveillance systems.

Patient monitoring and case surveillance provide ongoing, real-time data on the cascade of health services. However, investments will be needed in the security of data systems, their interoperability, and the capacity to use the data at national, district, community, and facility levels. This is aligned with the SDG Target 17.19 on country analytic and statistical capacity.

Third, it is important that no one is left behind with these data systems. This will require additional focus on integrating monitoring data from programs working with key populations and communities on prevention, and vital events data into a basic dataset that can be used for district level program decisions.

There are limitations to a reliance on routine data for both health care and reporting needs. Routine data are prioritized for sustainability but are also a necessity given limitations to funding. Routine surveillance should be supplemented by additional national and key population surveys, cohorts (valuable for estimates, incidence, and longitudinal cascade measures). Modeling is also critical for estimating indicators that are not easily measured, synthesising multiple source of data (routine data, surveillance data, survey data, demographic data) to better understand the epidemic and the impact of the HIV response. At the same time, surveys and modeling should not substitute for routine surveillance data, which will be the backbone for reporting needs.

Programs should also focus on routine program data because of its value for ongoing, everyday program decisions. The focus on routine data use in this paper puts surveillance data back into program settings where it can be used for millions of ongoing decisions by frontline health providers. Crucial to the incentives for quality reporting by health providers will be data feedback to all levels and use for program improvement. The sustainability of M&E systems will depend on data use in program settings for documented program improvement.

Routine surveillance data, including AIDS case surveillance, have been a critical component to mobilize the HIV care and prevention response to support health care workers, families and the community [19]. Over time, data generally have been taken out of the health care setting and community, with anonymous testing, national surveys, and a reliance on modelling. This paper suggests that routine data are part of implementation and that data are an important part of the public health intervention package of prevention, care, and data. Without routine data it is very difficult to have a public health or community response.

It is important that key surveillance data are put back into routine health care and community settings, as the SDGs regard data use as an intervention. This paper supports the use of ongoing, routine monitoring data for surveillance and program implementation. Person-centered, routine case and patient monitoring can contribute to the people-centered development focus of the SDGs, applied in a sustainable manner to data.

## Abbreviations

AIDS: acquired immune deficiency syndrome
CDC: Centers for Disease Control and Prevention
MDGs: Millennium Development Goals
M&E: monitoring and evaluation
NGO: non-governmental organization
PEPFAR: US President’s Emergency Plan for AIDS Relief
SDGs: Sustainable Development Goals
TB: tuberculosis
UN: United Nations
UNAIDS: Joint United Nations Programme on HIV/AIDS
WHO: World Health Organization
